# The emerging role of disease-associated microglia in Parkinson’s disease

**DOI:** 10.3389/fncel.2024.1476461

**Published:** 2024-11-05

**Authors:** Ritika R. Samant, David G. Standaert, Ashley S. Harms

**Affiliations:** ^1^Center for Neurodegeneration and Experimental Therapeutics, Department of Neurology, Heersink School of Medicine, University of Alabama at Birmingham, Birmingham, AL, United States; ^2^Aligning Science Across Parkinson’s (ASAP) Collaborative Research Network, Chevy Chase, MD, United States

**Keywords:** disease-associated microglia, neuroinflammation, Parkinson’s disease, Alzheimer’s disease, neurodegenerative diseases

## Abstract

Disease-associated microglia (DAM) are a subset of microglia that appear at various stages of central nervous system neurodegenerative diseases. DAM were identified using single-cell RNA sequencing within Alzheimer’s Disease (AD) where they were characterized by their unique localization near amyloid-β plaques and their phagocytic and lipid-metabolizing features. Unfortunately, activation and etiology of DAM are only understood within the context of AD where Triggering Receptor Expressed On Myeloid Cells 2 (TREM2), a receptor for amyloid-β, appears to be the key regulator in microglial transition to a DAM state. Despite this reliance on TREM2 in AD, DAM appear across other neurodegenerative diseases in which TREM2 may not be a critical player. This begs the question of if DAM are truly the same across all neurodegenerative diseases or if there exists a heterogeneity to DAM across neurodegenerative pathologies. Investigation into this critical gap in the field regarding DAM etiology and activation, as well as DAM function, could be delineated utilizing models of Parkinson’s disease (PD) to complement studies in models of AD. Though highly underexplored regarding DAM, PD with its pattern of protein aggregation-associated pathology like AD could serve as the spatiotemporal comparison against AD findings to ascertain the nature of DAM. The experimental vehicle that could guide the future of such investigation is the multi-omics model. With a compound approach focusing on exploring triggers for DAM at the chromatin or mRNA level and related protein output, it becomes possible to strongly characterize and firmly answer the question of what is a DAM.

## 1 Introduction

An important consequence of neuro-immune interaction is neuroinflammation, a pathological state where an inflammatory response takes place in the central nervous system (CNS; DiSabato et al., 2016). Neuroinflammation is best described as a shift in the homeostatic CNS environment mediated by both local responses and by interactions of the CNS with external and border-associated immune cell engagement (Paolicelli et al., 2022). Chronic neuroinflammation appears within the context of several neurodegenerative diseases including Alzheimer’s Disease (AD), Parkinson disease (PD), and Multiple Sclerosis (MS; Kempuraj et al., 2016). Persistent neuroinflammation is also a feature of aging, accompanied by increased blood–brain-barrier permeability and reduced glial signaling (Kempuraj et al., 2016).

Focusing on native CNS immune populations, 80% of the brain’s native immune cell population are microglia, traditionally viewed as the resident macrophages of the brain and spinal cord (Morimoto and Nakajima, 2019; Deczkowska et al., 2018). In steady-state maintenance of the brain and spinal cord, microglia support neural precursor cell proliferation. In neurodegenerative disease pathogenesis and aging, microglia shift into an activated state where they are heterogenous, functioning on a spectrum of wound-healing and wound-exacerbating (Crotti and Ransohoff, 2016; Deczkowska et al., 2018).

Accompanied by a shift in morphology from ramified to amoeboid, microglial activation—reactive microgliosis—is triggered by extracellular signaling or specific changes in the environment (Streit et al., 1999). These extracellular signals include foreign material, pathogens, and apoptotic by-products. In non-pathological states, this activation allows for microglial support in maintaining a homeostatic neuronal environment by phagocytosing debris and providing trophic support to neurons (Lull and Block, 2010). Microglial activation appears to increase with aging, but even so, microglial activation in neurodegenerative diseases appears to be a specific characteristic of the pathology (Lull and Block, 2010; Aguzzi et al., 2013). For example, within AD, the accumulation of amyloid plaques leads to microglial clustering and activation (Sasaki et al., 1997). Similarly activated microglia play a role in PD as indicated by foundational work conducted by McGeer et al. showing robust MHC-II staining within the substantia nigra (McGeer et al., 1988). Though these activated microglia appear to drive PD pathology through the release of inflammatory mediators—TNF-𝛼, IL-1β, and IL-6—activated microglia likely have other roles within the neurodegenerative environment and can participate in neural repair (Lull and Block, 2010; Colton, 2009). As such, there is still a limited understanding of what role microgliosis truly serves in neurodegenerative pathology. Traditional dichotomic confines of reactive microgliosis either driving the spread of pro-inflammatory proteins or fostering anti-inflammatory wound repair neglect one critical point: Microglial heterogeneity may be an inherent issue in functional studies.

The identification of “disease-associated microglia” (DAM) follows suit in this broader understanding of phenotypic and therefore functional variety in microglia as framed by disease, development, and aging within the context of neurodegenerative disease. DAM are a subset of microglia that appear to be activated in a variety of neurodegenerative pathological conditions (Sobue et al., 2021; Keren-Shaul et al., 2017).

**Figure 1:**
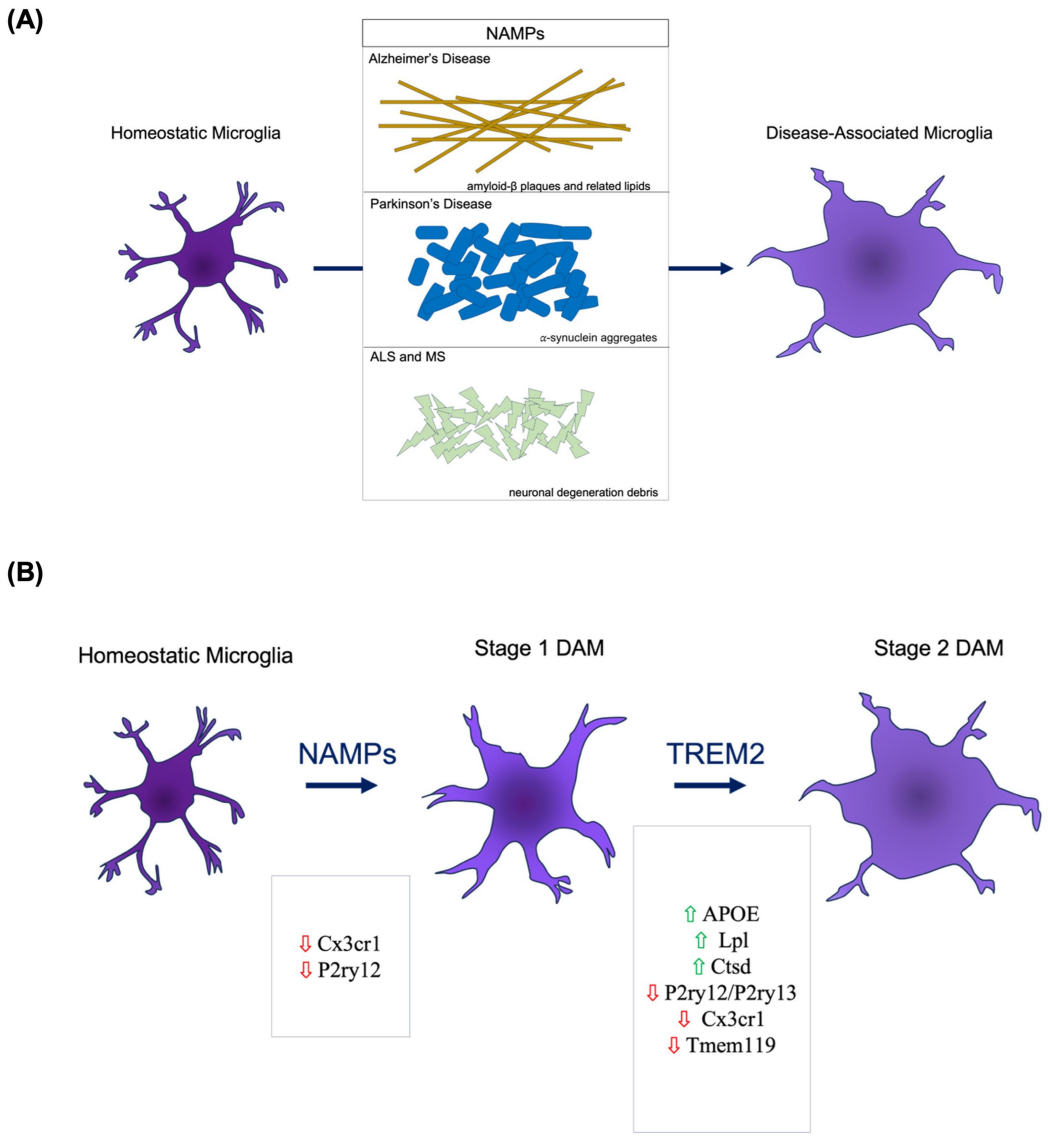
DAM Etiology and Activation. **(A)** The presence of neurodegeneration-associated molecular patterns (NAMPs) serves as key molecular triggers of pattern recognition receptors on microglia that trigger DAM activation pathways. **(B)** The DAM activation occurs through a two-stage process. The transition from homeostatic to Stage 1 involves decreased presence of homeostatic microglial markers. The Stage 1 to Stage 2 DAM transition necessitates TREM2 signaling.

Thought to have unique biological properties, DAM localize at the physical and biological interfaces of the brain and peripheral immune system near sites of injury or degeneration where apoptosis has occurred; a location that gives them a central role in neuro-immune modulation (Deczkowska et al., 2018). This aligns with transcriptional signatures of DAM within the context of AD where DAM demonstrate a lipid-metabolizing and phagocytic activation state (Keren-Shaul et al., 2017). Though largely characterized primarily in AD thus far, it seems that DAM emerge within a neuroinflammatory context in response to neurodegeneration-associated molecular patterns (NAMPs; Deczkowska et al., 2018; Figure 1A). It is because of this close connection to injury and neuroinflammation that DAM hold potential as an important target for therapeutic modification in neurodegenerative diseases and aging.

In this mini-review, we explore this critical gap in current DAM literature, present a series of critical points for consideration regarding future investigation, and make a case for PD as the next frontier for DAM exploration due to its sister-like similarity to AD.

## 2 The historical emergence of DAM

In foundational work conducted by Keren-Shaul et al., DAM were first identified in AD, ALS, and aging through single-cell RNA sequencing (scRNA seq), immunohistochemistry (IHC), and single molecule fluorescent *in situ* hybridization (smFISH; Keren-Shaul et al., 2017). scRNA seq showed that DAM appeared as a distinct state from homeostatic microglia when exploring:

(1) The expression of classic homeostatic microglial genes like Cystatin C (Cst3) and Hexosaminidase Subunit B (Hexb), (2) the decrease of other homeostatic microglial genes like Purinergic Receptor P2Y (P2ry12/P2ry13), C-X3-C Motif Receptor 1 (Cx3cr1) and Transmembrane Protein 119 (Tmem119), (3) the distinct increase in lipid metabolizing and phagocytic genes like Alipoprotein E (APOE), lipoprotein lipase (Lpl) and other known AD factors like Cathepsin D (Ctsd), and Triggering Receptor Expressed On Myeloid Cells 2 (TREM2; Keren-Shaul et al., 2017).

IHC staining for CD11c and IBA-1, a classic microglial marker, paired against stained Aβ plaques showed a population of activated microglia near Aβ-plaques in AD. smFISH showed a significantly increased expression of DAMs-specific genes (Csf1 and Lpl) in the activated microglial population that localizes near amyloid-β plaques in AD (Keren-Shaul et al., 2017).

Interestingly, DAM exist beyond the bounds of defined clinical neurodegenerative disease states. Exploration of isolated intracranial injection of neuronal apoptotic bodies in naïve mice have show a resulting DAM phenotype within 16 h independent of AD or PD pathology (Krasemann et al., 2017).

These findings reveal a critical feature of DAM: localization at the physical and biological interfaces of the brain and peripheral immune system near sites of injury or degeneration and near sites of apoptosis; a location that gives DAM the potential central role in neuro-immune modulation.

With a localization proximal to sites of injury and degeneration and a genetic profile outlining lipid-metabolizing and phagocytic properties, DAM likely engage in clearance of neurodegenerative byproducts. This implies a neuroprotective role for the DAM through both containment and clearance of injury. This is highly speculatory, as no study to date has been done to definitively prove DAM are helpful via pathogen clearance. Regardless, with a close connection to injury and neuroinflammation DAM hold potential as an important target for therapeutic modification in neurodegenerative diseases and aging.

## 3 DAM in neurodegeneration

DAM etiology and activation is largely understood only within AD. In AD, DAM emerge through a two-step transition process from the homeostatic state to a Stage 1 intermediate to a Stage 2 DAM (Figure 1B). NAMPs appear to be a potential trigger for the homeostatic to Stage 1 transition (Deczkowska et al., 2018). Of the genes expressed throughout the DAM initiation process, TREM2 appears to be central in regulating the Stage 1 to Stage 2 transition (Deczkowska et al., 2018). Following Stage 2, maintenance of DAM upregulated gene pathways is necessary. APOE is an upregulated DAM-associated gene and a suspected molecule involved in the autocrine or paracrine loop sustaining the DAM activation (Song and Colonna, 2018). Specifically, it is the TREM2-APOE pathway that appears to facilitate the shift from homeostatic to DAM following microglial phagocytosis of apoptotic neuronal bodies (Poliani et al., 2015). When modifying this pathway via reduction of TREM2, there is a decreased clearance of apoptotic neurons (Takahashi et al., 2005). Complementarily, APOE deletion and deficiency leads to a reduced DAM signature in neurodegenerative and acute injury models (Song and Colonna, 2018). When pairing these findings of DAM activation and resultant apoptotic neuronal debris clearance, there emerges an implication that DAM could play a neuroprotective role through prevention of inflammatory relays via NAMPs left from apoptosis.

It is important to note that the quality of DAM as exacerbating or healing within the context of AD is not uniform. That said, the effects of TREM2 deletion vary along progression stage of the disease implying a disease-state-dependent role of DAM in AD (Jay et al., 2017).

Curiously, though AD implicates TREM2 as necessary for the transition to DAM, DAM exist in other diseases where TREM2 is not genetically linked like ALS and MS. ALS is a neurodegenerative disease characterized by the progressive degeneration of upper and lower motor neurons. A single-cell analysis of CD45+ cells from the spinal cord of a transgenic mouse model of ALS highlights a microglial population, with a genetic expression adjacent to AD DAM, that increases over the course of ALS (Keren-Shaul et al., 2017). TREM2 appears to be upregulated in ALS DAM though it has not been confirmed if TREM2 acts similarly to its role in AD as a phenotypic trigger from Stage 1–Stage 2 DAM (Xie et al., 2022). Additionally, while DAM have been spatially mapped in AD, specifics about locations in ALS and what this may imply for disease progression require further investigation.

In concurrent work from Krasemann et al., DAM were outlined with another name, microglial neurodegenerative phenotype (MGnD; Krasemann et al., 2017). In this study, MGnD were first classified using AD, ALS, and MS models. Multiple sclerosis (MS) is a neurodegenerative disease marked by chronic inflammation and the presence of CNS lesions comprised of glial cells and demyelination products. Beyond bearing a similar genotypic profile that led to defining DAM/MGnD, the specifics about DAM in MS have yet to be explored. The general understanding of activated microglia within the MS lesions and the lipid-metabolizing nature of DAM, as outlined within AD, could point to a potentially negative role of DAM in exacerbating or partaking in the demyelination that is a hallmark of MS.

Though the understanding of DAM is still in its infancy, one thing becomes increasingly clear—the characterization of DAM is largely limited to AD. The link between TREM2 and DAM progression in AD is strongly outlined in a way that remains unparalled by other neurodegenerative diseases, though recent studies have shown TREM2 to be associated with PD and MS pathology, making it a promising marker to explore (Guo et al., 2019, Rayaprolu et al., 2013, Cignarella et al., 2020). Thus, without a uniform notable mediator across all neurodegenerative diseases, two critical implications emerge:

1. DAM in AD must be intrinsically different than in other neurodegenerative diseases, as TREM2 is not a major player or risk factor.
2. Grouping all neurodegenerative diseases with this microglial phenotype as “DAM” may be too macroscopic of an approach.

These implications require diligent investigation of DAM in other neurodegenerative diseases to better characterize and potentially endeavor to sub-classify DAM. A strong starting place for said investigation with ironically limited exploration of DAM is PD.

## 4 DAM in PD

### 4.1 Comparisons between PD and AD

Given the parallels between PD and AD—chronic long-lived diseases marked by pathological protein misfolding and accumulation—it seems likely that the microglial subsets identified in AD functionally exist in PD. Both neurodegenerative diseases follow a temporal progression with corresponding misfolded protein aggregation. Though misfolded amyloid-β form extracellular aggregates and misfolded 𝛼-synuclein comprise intracellular Lewy Bodies, functionally, 𝛼-synuclein similar to amyloid-β leads to a local environment ripe with NAMPs that drive a microglial shift into a reactive state (Zhang et al., 2005). Due to research studies largely modeling PD through acute measures, the effects and full understanding of sustained 𝛼-synuclein NAMPs and microglial activation in PD are limited. Within other pathological neurodegenerative disease-states like PD, where APOE and TREM2 do not appear to be implicated in disease risk or pathogenesis, it is likely that other implicated genes drive an analogous development of a functionally similar DAM. Within PD, genome wide association studies and related analysis point to P2RY12 (Purinergic Receptor P2Y, G-Protein Coupled, 12) as a potential causative player in PD that could engage in DAM activation (Andersen et al., 2021). P2RY12 is a marker for homeostatic microglia and is a chemotactic receptor guiding microglia to sites of injury for repair (Lou et al., 2016).

However, harkening back to the implications discussed prior, because AD and PD are inherently different with unique disease etiology and non-overlapping genetic markers, there arises a set of critical questions:

1. Are all DAM subsets the same across neurodegenerative disease?
2. With the possibility for additional heterogeneity within DAM, should the field look towards establishing disease-related subsets for DAM given their potential different functional consequences?
3. What needs to be investigated to provide a clear definition of what is a DAM?

### 4.2 Presence of DAM in PD

⍺-synuclein induced neuroinflammation has been shown to heighten neurodegeneration. Though the specifics of what role microglia play in this neuroinflammation still remain unclear, recent studies have begun exploring the presence of DAM in PD. Schonhoff et al. lays the groundwork for DAM in PD with the conclusion that DAM in PD do exist. Consistent with the literature, the DAM were identified via scRNA for their expression of Cystatin F (Cst7), Lpl, and APOE (Schonhoff et al., 2022). This conclusion is reached while also exploring border-associated macrophages (BAM), which serve as the antigen presenting cells that trigger CD4 T-cell mediation of *α*-synuclein related neuroinflammation. Increased *α*-synuclein expression correlated with a two-fold increase in MHC-II and PD-L1 expression in DAM cohorts and increased BAM expression where BAM were identified in spatial proximity to T cells in post-mortem PD brains (Schonhoff et al., 2022). Interestingly, scRNA sequencing revealed the presence of microglial DAM genes expressed in the PD animal model aligned with DAM genes expressed in the AD animal model characterized by Keren-Shaul and revealed a new classification of border associated macrophages, termed disease-associated BAMs (DaBAMs).

To understand DAM in a spatiotemporal context, methods to image DAM in models of AD have been conducted using immunofluorescence, specifically single molecule fluorescence *in situ* hybridization (Keren-Shaul et al., 2017; Deczkowska et al., 2018). Further investigation exploring the spatiotemporal placement of DAM against the 𝛼-synuclein aggregation throughout PD progression would progress the understanding of the role of DAM in PD.

Considering the recent BAM studies and the understood neuroinflammatory environment of PD, the PD-variation of DAM does exist. The role of DAM in PD requires further exploration but postulating based on what is understood about DAM within the AD context could provide insight into avenues for harnessing therapeutic potential of DAM in PD. Though distinct disease-states with differing etiologies and pathologies, these disorders overlap in clinical and neuropathological fundamental features like misfolded protein aggregates making a case for a translational relationship of the properties of DAM in AD to the properties of DAM in PD (Perl et al., 1998).

As such, it is possible that DAM in PD engage in phagocytic clearance of the 𝛼-synuclein Lewy bodies accumulated in the SNpc—something in principle that may be neuroprotective if employed therapeutically at the appropriate spatiotemporal point. If engaged too early or executed in a widespread manner, the effects could prove detrimental in exacerbating the progression of PD.

### 4.3 PD modeling limitations and future directions

Though the presence of DAM in PD appears to be corroborated per ongoing and past investigation, it is important to note limitations in both PD modeling and in transcriptomics-focused approaches to addressing the functionality of DAM.

The complex nature of PD and the unknown etiology entail a variety of animal models to model and test competing theories of disease pathogenesis. Neurotoxin-based approaches involve acute or chronic exposure of animal models to 6-hydroxy dopamine (6-OHDA), 1-methyl-4phenyl-1,2,3,6-tetrahydropyridine (MPTP), and agrochemicals such as rotenone; genetic-based models of PD include viral vector-mediated models, wherein adeno-associated viral vectors (AAV) expressing human 𝛼-synuclein are injected into the substantia nigra, and transgenic models derived from genes implicated in PD like SNCA, LRKK2, PINK1, and PRKN; and, alterations regarding SNCA through mutation or through introduction of *α*-synuclein pre-formed fibrils have varied effects that include nigrostriatal neurodegeneration, α-synuclein aggregation, and motor deficits (Stoker et al., 2018; Garcia et al., 2022).

The resulting pathology of the varied models vary per species used and related environment, thus, making a combination of models ideal for studying the complex relationship between genetics and the environment that frame PD pathology. Current understanding of DAM in PD has come from PD pathology induced by 𝛼-synuclein via AAV transduction (Schonhoff et al., 2022) in which neuroinflammation precedes neurodegeneration, but investigation in other models and in post-mortem human PD tissue could yield a more nuanced understanding of DAM (Garcia et al., 2022).

Beyond the nuance of disease modeling and experimental design lies a deeper question, iterated prior, regarding DAM functionality: What needs to be investigated to provide a clear understanding of what DAM are?

Transcriptomics have been used to assess the presence and functionality of DAM within PD and in other neurodegenerative diseases. But transcriptomics fail to capture the complex presentation of cellular function as mediated by actual protein presence and quantity as shown by multiple studies (Rogers et al., 2008; Chen et al., 2002; Pascal et al., 2008). In AD, the proteomics approach has been used alongside transcriptomics to provide rationale for grouping DAM into inflammatory response related subsets (Rangaraju et al., 2018). As such, spatially integrating proteomics-based approaches in investigating DAM in PD would enhance the field’s understanding and provide yet another means of comparison between PD and AD. While incredibly complex, a multi-omics approach could be the right vehicle for DAM exploration.

## 5 Conclusion

Though a newer microglial classification, there is a pertinent gap in the field that needs to be addressed such that DAM can be functionally understood. With varied etiology and activation mechanisms in relation to the disease microenvironment it stems from, DAM may not truly all be the same. Viewing them as such may be a gross oversimplification that hinders nuanced investigation of harnessing therapeutics that target DAM. As of now, in AD and aging, the role of DAM leans neuroprotective but only conditionally based on time and location in the CNS. PD can be an excellent disease setting for additional DAM investigation, especially with regards to the time and ⍺-synuclein pathology. A sister to AD in terms of disease progression, investigating the spatiotemporal properties of DAM in PD could provide a strong baseline for comparison from which to conclude whether or not DAM are identical in terms of mechanisms performed across neurodegenerative diseases. This would shed light on whether DAM are functionally identical from a greater neuroprotective or neurodegenerative standpoint. These functional studies are necessary before potential disease modifying therapeutic targeting to DAM are even considered for investigation. A strong contender for assessing DAM functionality lies in a multi-omics model. With the variety in known etiologies and widely used pathogenesis triggers for PD including genetic, environmental, and *α*-synuclein fibril seeding, it becomes pertinent to consider technologies such as cellular indexing of transcriptomes and epitopes sequencing (CITE-seq) or assay for transposase-accessible chromatin (scATACseq) in a longitudinal investigation across these varying models. The hope is that such a compound approach would allow for a clearer picture to emerge of what exactly triggers DAM at the transcriptional or chromatin level and if there is consistency in DAM functionality across time at the protein level. The addition to imaging to such a multi-omics approach would then paint a spatiotemporal picture of DAM in neurodegenerative disease and identify disease specific heterogeneity. When paired against human PD tissue, this approach has the potential to elucidate what DAM really are with the potential to examine if PD models are consistent.
